# Structured Reporting of Computed Tomography and Magnetic Resonance in the Staging of Pancreatic Adenocarcinoma: A Delphi Consensus Proposal

**DOI:** 10.3390/diagnostics11112033

**Published:** 2021-11-03

**Authors:** Vincenza Granata, Giovanni Morana, Mirko D’Onofrio, Roberta Fusco, Francesca Coppola, Francesca Grassi, Salvatore Cappabianca, Alfonso Reginelli, Nicola Maggialetti, Duccio Buccicardi, Antonio Barile, Marco Rengo, Chandra Bortolotto, Fabrizio Urraro, Giorgia Viola La Casella, Marco Montella, Eleonora Ciaghi, Francesco Bellifemine, Federica De Muzio, Ginevra Danti, Giulia Grazzini, Carmelo Barresi, Luca Brunese, Emanuele Neri, Roberto Grassi, Vittorio Miele, Lorenzo Faggioni

**Affiliations:** 1 Division of Radiology, Istituto Nazionale Tumori IRCCS Fondazione Pascale—IRCCS di Napoli, 80131 Naples, Italy; v.granata@istitutotumori.na.it; 2 Department of Radiology, Santa Maria di Ca’ Foncello Hospital, 31100 Treviso, Italy; giovannimorana@gaslini.org; 3 Department of Radiology, University Hospital G.B. Rossi, University of Verona, 37134 Verona, Italy; mirko.donofrio@univr.it; 4 Medical Oncology Division, Igea SpA, 80013 Napoli, Italy; 5 Department of Radiology, IRCCS Azienda Ospedaliero-Universitaria di Bologna, 40138 Bologna, Italy; francesca_coppola@hotmail.com; 6 Italian Society of Medical and Interventional Radiology (SIRM), SIRM Foundation, Via della Signora 2, 20122 Milan, Italy; francescagrassi1996@gmail.com (F.G.); antonio.barile@univaq.it (A.B.); emanueleneri1@gmail.com (E.N.); roberto.grassi@unicampania.it (R.G.); vmiele@sirm.org (V.M.); 7 Division of Radiology, Università degli Studi della Campania Luigi Vanvitelli, 80125 Naples, Italy; salvatore.cappabianca@unicampania.it (S.C.); alfonso.reginelli@unicampania.it (A.R.); fabrizio.urraro@unicampania.it (F.U.); giorgialacasella@glose.it (G.V.L.C.); marco.montella@unicampania.it (M.M.); 8 Section of Radiodiagnostic, DSMBNOS, Aldo Moro University, 70124 Bari, Italy; n.maggialetti@gmail.com; 9 Imaging Department, San Paolo Hospital, 17100 Savona, Italy; buccicardiduccio@hotmail.com; 10 Department of Applied Clinical Sciences and Biotechnology, University of L’Aquila, 67100 L’Aquila, Italy; 11 Department of Radiological Sciences, Oncology and Pathology, Sapienza University of Rome I.C.O.T. Hospital, Via Franco Faggiana, 1668, 04100 Latina, Italy; marco.rengo@gmail.com; 12 Department of Radiology, I.R.C.C.S. Policlinico San Matteo Foundation, 27100 Pavia, Italy; chandra.bortolotto@gmail.com; 13 Exprivia SpA, 70056 Bari, Italy; eleonora.ciaghi@exprivia.com (E.C.); Francesco.Bellifemine@exprivia.com (F.B.); 14 Department of Medicine and Health Sciences “V. Tiberio”, University of Molise, Via Francesco De Sanctis 1, 86100 Campobasso, Italy; demuziofederica@gmail.com (F.D.M.); lucabrunese@libero.it (L.B.); 15 Division of Radiology, Azienda Ospedaliera Universitaria Careggi, 50134 Florence, Italy; ginevra.danti@gmail.com (G.D.); grazzini.giulia@gmail.com (G.G.); 16 Diagnostic Imaging Section, Department of Medical and Surgical Sciences & Neurosciences, Siena University Hospital, 53100 Siena, Italy; carminev80@alice.it; 17 Department of Translational Research, University of Pisa, 56126 Pisa, Italy; lfaggioni@sirm.org

**Keywords:** radiology report, structured report, pancreatic adenocarcinoma, computed tomography, magnetic resonance imaging

## Abstract

Background: Structured reporting (SR) in radiology has been recognized recently by major scientific societies. This study aims to build structured computed tomography (CT) and magnetic resonance (MR)-based reports in pancreatic adenocarcinoma during the staging phase in order to improve communication between the radiologist and members of multidisciplinary teams. Materials and Methods: A panel of expert radiologists, members of the Italian Society of Medical and Interventional Radiology, was established. A modified Delphi process was used to develop the CT-SR and MRI-SR, assessing a level of agreement for all report sections. Cronbach’s alpha (Cα) correlation coefficient was used to assess internal consistency for each section and to measure quality analysis according to the average inter-item correlation. Results: The final CT-SR version was built by including n = 16 items in the “Patient Clinical Data” section, n = 11 items in the “Clinical Evaluation” section, n = 7 items in the “Imaging Protocol” section, and n = 18 items in the “Report” section. Overall, 52 items were included in the final version of the CT-SR. The final MRI-SR version was built by including n = 16 items in the “Patient Clinical Data” section, n = 11 items in the “Clinical Evaluation” section, n = 8 items in the “Imaging Protocol” section, and n = 14 items in the “Report” section. Overall, 49 items were included in the final version of the MRI-SR. In the first round for CT-SR, all sections received more than a good rating. The overall mean score of the experts was 4.85. The Cα correlation coefficient was 0.85. In the second round, the overall mean score of the experts was 4.87, and the Cα correlation coefficient was 0.94. In the first round, for MRI-SR, all sections received more than a good rating. The overall mean score of the experts was 4.73. The Cα correlation coefficient was 0.82. In the second round, the overall mean score of the experts was 4.91, and the Cα correlation coefficient was 0.93. Conclusions: The CT-SR and MRI-SR are based on a multi-round consensus-building Delphi exercise derived from the multidisciplinary agreement of expert radiologists in order to obtain more appropriate communication tools for referring physicians.

## 1. Introduction

Pancreatic cancer accounts for almost as many deaths (466,000) as cases (496,000) because of its poor prognosis and is the seventh leading cause of cancer death in both sexes. Rates are from 4-fold to 5-fold higher in higher Human Development Index (HDI) countries, with the highest incidence rates in Europe, Northern America, and Australia/New Zealand [1]. Both incidence and mortality rates either have been stable or have slightly increased in many countries, likely reflecting the increasing prevalence of obesity, diabetes, and alcohol consumption, although improvements in diagnostic and cancer registration practices may also be in play in some countries [1,2,3,4]. Given that the rates of this disease are rather stable relative to the declining rates of breast cancer, it has been projected that pancreatic cancer will surpass breast cancer as the third leading cause of cancer death by 2025 in a study of 28 European countries [1].

Pancreatic ductal adenocarcinoma (PDAC) is a challenge for a multidisciplinary oncology team. Although many patients have locally advanced disease at diagnosis, the only curative treatment is surgery, and systemic chemotherapy is usually the key therapy [5,6,7,8,9]. The multidisciplinary team should make the choice concerning the resectability of pancreatic cancer following the acquisition of a complete staging [10,11]. Computed tomography (CT) has grown to be the tool of choice in the preoperative diagnosis guiding treatment planning, as well as during follow-up [12,13]. Several researchers considered magnetic resonance imaging (MRI) to be equivalent to CT in detecting and staging. However, recent evidence recommends the addition of MRI as a diagnostic integration to identify lesions undetected by CT as well as the presence of liver metastases [14,15]. MRI is a useful diagnostic tool in oncologic patients since this offers morphological data by T2-weighted (W) and T1-W sequences, and functional data by diffusion-weighted imaging (DWI) and dynamic contrast enhanced (DCE)-MRI, as well as new tools such as blood oxygenation-level dependent (BOLD) sequences [14,15].

Imaging evaluation plays a central and primary role in the initial decision making process of patients with PDAC. There are, however, limitations in the current reporting of these imaging studies. These include variability of the descriptive terminology that attempts to define disease extent and incomplete documentation of disease sites which may affect prognosis and adversely affect treatment planning by surgeons, and medical and radiation oncologists [16]. An effective communication of imaging data to referring physicians is crucial for patient care. Radiology reports are traditionally created as free-text reports (FRT) in narrative language. However, inconsistencies with regard to content, style, and presentation can hamper data transfer and reduce the clarity of the reports, which can in turn negatively affect the extraction of the required key information by the referring physician [16,17,18,19,20,21,22]. Therefore, FRT should be shifted toward structured reports (SRs). The use of templates in SR provides a checklist as to whether all relevant items for a specific procedure are addressed. Moreover, thanks to this “structure”, SR allows the correlation of radiological data with other key clinical features, guiding to a precise diagnosis and personalized medicine [23,24,25,26,27,28]. 

Despite the evident improvements, SRs have not yet become approved in the radiological routine. The main reasons are the current lack of usable templates and the minimal availability of software solutions [19]. Therefore, the Italian Society of Medical and Interventional Radiology (SIRM) elaborated an Italian warehouse of SR templates that can be freely accessed by all SIRM members, with the purpose of the routine use in a clinical setting [29]. 

For PDCA patients, the aim of treatment should be curative when possible. The tumor size, location within the pancreas, local extent which may involve surrounding vessels, and the presence of metastatic lesions should be assessed before planning treatment. All these features should be reported in a radiological template, and therefore an adequate communication between the radiologist and multidisciplinary group is required. To improve this communication and meet the needs of clinicians, the aim of this study is to propose an SR template for PDAC based on CT and MRI study in the systematic reporting of neoplasm findings at the staging phase.

## 2. Materials and Methods 

### 2.1. Panel Expert

A multi-round consensus-building Delphi exercise, and subsequent extensive discussion between expert radiologists, was completed to create comprehensive focused SR templates for CT and MRI during the staging phase of patients with PDAC. 

A SIRM radiologist, an expert in abdominal imaging, created the first drafts of the SRs. A working team of 20 experts was set up, with members from the Italian College of Gastro-enteric Radiologists and of Diagnostic Imaging in Oncology Radiologists from SIRM. Their aim was to revise the initial drafts iteratively, with the objective of reaching a final consensus on SRs.

### 2.2. Selection of the Delphi Domains and Items

All the experts reviewed literature data on the main scientific databases, including PubMed, Scopus, and Google Scholar, to assess papers on pancreatic cancer imaging and structured radiology reports from December 2000 to August 2021. All panelists reviewed the full text of the selected studies, and each of them shared the list of Delphi items via emails and/or teleconferences. 

The CT-SR was divided into four sections: (a) Patient Clinical Data, (b) Clinical Evaluation, (c) Imaging Protocol, and (d) Report. A dedicated section of significant images was added as part of the report. 

The MRI-SR was divided into four sections: (a) Patient Clinical Data, (b) Clinical Evaluation, (c) Imaging Protocol, and (d) Report. A dedicated section of significant images was added as part of the report. 

Two Delphi rounds were performed. During the first round, each panelist independently contributed to refining the SR drafts by means of online meetings or email exchanges. The level of panelists’ agreement for each SR models was tested in the second Delphi through a Google Form questionnaire shared by email. Each expert expressed individual comments for each specific template section using a five-point Likert scale (1 = strongly disagree, 2 = slightly disagree, 3 = slightly agree, 4 = generally agree, 5 = strongly agree).

After the second Delphi round, the last version of the SRs was generated on the dedicated RSNA website (radreport.org) using a T-Rex template format, in line with IHE (Integrating the Healthcare Enterprise) and the MRRT (Management of Radiology Report Templates) profiles, accessible as open-source software, with the technical support of Exprivia (Exprivia SpA, Bari, Italy). These determine both the format of radiology report templates (using version 5 of HyperText Markup Language (HTML5)) and the transporting mechanism to request, retrieve, and stock these schedules [30]. The radiology report was structured using a series of “codified queries” integrated in the T-Rex editor’s preselected sections [30]. 

### 2.3. Statistical Analysis

Answers from each panelist were exported in Microsoft Excel^®^ format for ease of data collection and statistical analysis.

All ratings of panelists for each section were analyzed with descriptive statistics measuring the mean score, the standard deviation, and the sum of scores. A mean score of 3 was considered good and a score of 4 excellent. 

To measure the internal consistency of the panelist ratings for each section of the report, a quality analysis based on the average inter-item correlation was performed with Cronbach’s alpha (Cα) correlation coefficient [31,32]. An alpha coefficient (α) ≥ 0.9 was considered excellent, α ≥ 0.8 good, α ≥ 0.7 acceptable, α ≥ 0.6 questionable, α ≥ 0.5 poor, and α < 0.5 unacceptable. However, in the iterations an α of 0.8 was considered a reasonable goal for internal reliability.

The data analysis was performed using the Statistic Toolbox of MATLAB (The MathWorks, Inc., Natick, MA, USA).

## 3. Results

### 3.1. Structured Report 

The final CT-SR (Supplementary Materials) version was built by including n = 16 items in the “Patient Clinical Data” section, n = 11 items in the “Clinical Evaluation” section, n = 7 items in the “Imaging Protocol” section, and n = 18 items in the “Report” section. Overall, 52 items were included in the final version of the CT-SR. 

The final MRI-SR (Supplementary Materials) version was built by including n = 16 items in the “Patient Clinical Data” section, n = 11 items in the “Clinical Evaluation” section, n = 8 items in the “Imaging Protocol” section, and n = 14 items in the “Report” section. Overall, 49 items were included in the final version of the MRI-SR. 

For both templates, only the report section must be compiled, and all other sections are optional.

The “Patient Clinical Data” section included patient anthropometric data, previous or family history of malignancies, including pancreatic cancer, risk factors, including genetic mutations, and predisposing diseases. In this section, we included the item “Allergies” to drug or no drug and contrast medium.

The “Clinical Evaluation” section collected previous examination results, a genetic panel, results of histopathological examination on biopsy specimen, carbohydrate antigen 19.9 (Ca 19.9) level, carcinoembryonic antigen (CEA) level, blood count, serum creatinine, liver function, and clinical symptoms. 

The “Imaging Protocol” section for CT-SR included data on the equipment used, including data on the reconstruction algorithm and slice thickness. In addition, we collected data on contrast study protocol, including data on the contrast study phase, as well as data concerning the contrast medium, and ongoing adverse events. 

The “Imaging Protocol” section for MRI-SR included data on the scanner brand and model, protocol details as conventional or abbreviated, and sequences. In addition, we collected data on contrast study protocol, including data on the contrast study phase, as well as data concerning the contrast medium. 

The “Report” section included data on:

1. Lesion: tumor visible or not visible and indirect signs; size; structure; site; vascularity.

2. Arteries: normal or variant anatomy; atherosclerotic; presence of vessel involvement; distance between celiac trunk and infiltrated hepatic artery >5 mm.

3. Veins: normal or variant anatomy; thrombosis (neoplastic or not neoplastic); presence of vein involvement; for superior mesenteric vein, longitudinal extent of infiltration >20 mm and tumor involvement of first jejunal loop.

In addition, in this section, we included data on biliary ducts, posterior lamina, loco-regional diffusion (including stomach, spleen, treits, liver, etc.), node stage, and for MRI-SR, liver metastases; for CT-SR, metastases stage (including liver, bone, lung, etc.), peritoneal carcinomatosis, as well as the presence of incidental radiological findings, including acute pancreatitis and pulmonary embolism.

### 3.2. Consensus Agreement

In the first round, as reported in Table 1, for CT-SR, all sections by 20 panelists received more than a good rating. The overall mean score of the experts was 4.85 (range 2–5). The Cα correlation coefficient was 0.85 for the CT staging structured report and the sum of scores was 1844 (92.20 ± 5.57).

Table 2 reports single score and sum of scores of 20 panelists for the MR staging structured report in the first round. In the first round, as reported in Table 2, all sections received more than a good rating. The overall mean score of the experts was 4.73 (range 1–5). The Cα correlation coefficient was 0.82 and the sum of scores for the MR structured report was 1798 (89.90 ± 6.64).

Table 3 reports single score and sum of scores of 20 panelists for the CT staging structured report in the second round: all sections received more than a good rating. The overall mean score of the experts was 4.87 (range 3–5), the Cα correlation coefficient was 0.94, and the sum of scores was 1850 (92.50 ± 4.03).

In the second round for the MR structured report, as reported in Table 4, all sections by 20 panelists received more than a good rating. The overall mean score of the experts was 4.91 (range 3–5), the Cα correlation coefficient was 0.93, and the sum of scores for the MR structured report was 1108 (93.35 ± 3.28). 

For both the CT and MR pancreas structured report, between the first and second round, a major agreement was reached among the 20 panelists highlighted by the increase of Cα correlation coefficient, overall mean score, and sum of scores. 

## 4. Discussion

The advantages of SR are centered around 11 major themes: accuracy/quality, retrievability, accessibility, automatization, facilitation of workflows, keeping the electronic patient record (EPR) up to date, teleradiology, information exchange between medical centers, ergonomics of the radiologist and the referring physician, financial benefits, and education [25,26]. Accuracy and quality assurance of the report were important topics. SR would encourage radiologists to employ a specific lexicon and therefore keep them from hiding behind vague and verbose reports [26]. A tiered approach to SR has been described [26]. At its basic level, SR should be organized with headings, such as clinical history, indication, technique, findings, and impression. The next tier is where the “findings” section is organized with subheadings, such as the various organs imaged. At the highest tier, the SR has all of the previously mentioned features and uses a standardized language based on a universally accepted lexicon [26]. The present SRs are based on standardized terminology and structures, features required in order to adhere to diagnostic–therapeutic recommendations and enrolment in clinical trials, reducing any ambiguity that may arise from non-conventional language and enable better communication between radiologists and clinicians so that they are third-level reports [26].

According to our knowledge, a similar work was already carried out by a multi-institutional group of 15 experts that included radiologists, hepatopancreatobiliary surgeons, and gastroenterologists, composed of members of the Society of Abdominal Radiology and the American Pancreatic Association, who convened a consensus conference during the annual American Pancreatic Association meeting (Chicago, November 2011) [16]. A draft template including the most appropriate findings chosen from the available templates based on the state of knowledge and available pertinent literature was developed by consensus during the meeting. A final draft was prepared by the lead author and sent to all participants for review, comments, and approval. Unlike the template presented in 2014 [16], the present templates were based on a multi-round consensus-building Delphi exercise following in-depth discussion between expert radiologists in gastro-enteric and oncological imaging. In addition, in this project we promoted two templates, one for CT and one for MRI, which, unlike what is promoted by Al-Hawary [16], are composed of a mandatory section which is “the report section” and three optional sections. In fact, the final CT-SR version was built by 52 items, including n = 16 items in the “Patient Clinical Data” section, n = 11 items in the “Clinical Evaluation” section, n = 7 items in the “Imaging Protocol” section, and n = 18 items in the “Report” section. The final MRI-SR version was built by 49 items, including n = 16 items in the “Patient Clinical Data” section, n = 11 items in the “Clinical Evaluation” section, n = 8 items in the “Imaging Protocol” section, and n = 14 items in the “Report” section. For both CT and MR pancreas SR, between the first and second round, a major agreement was reached among the 20 panelists highlighted by the increase of Cα correlation coefficient, overall mean score, and sum of scores. 

We think that this result is due to the awareness of the need to identify the essential features to be reported in a radiological report and, from another point of view, from the idea that today there is a need to integrate clinical data with radiological data. Although the present template may seem long and difficult and therefore slow down the workflow of a radiologist, it is necessary to emphasize that only the report section is mandatory, while the others are optional. Furthermore, considering that not all data may be available to the radiologist, they are open fields that can also be filled in a later time. In addition to this, the possibility of connecting this template with the patient’s electronic file allows for an automatic import of the available data. Beyond the idea of the scrupulousness of these reports, we believe it is useful to also fill in the optional fields; in fact, the “Patient Clinical Data” section included patient anthropometric data, previous or family history of malignancies, risk factors and predisposing diseases, family history of pancreatic cancer, hereditary syndromes, and other genetic mutations. The “Clinical Evaluation” section collected previous examination results, a genetic panel, results of histopathological examination on biopsy specimen, carbohydrate antigen 19.9 (Ca 19.9) level, carcinoembryonic antigen (CEA) level, blood count, serum creatinine, liver function, and clinical symptoms. These data could create the basis of a large database, allowing not only for the carrying out of epidemiological statistical analysis, but they could be used to build a radiomics model by combining radiological features and clinical data [33,34,35,36,37,38,39]. In this context, the added value of genomic data could be used to develop a model of radiogenomics, which was helpful regarding the highest level of personalized risk stratification and the advanced precision medicine process [40,41,42,43,44,45].

With regard to the “Imaging Protocol” section, revealing the examination technique, not only within one’s own department, but also with radiology departments of other centers, rejoins to a double reason: the standardization and the optimization of the study protocols. For example, during oncological follow-up, different acquisition parameters such as different segmentation algorithms are crucial elements that can lead to variability in volumetric assessment. Thus, for CT, slice thickness and other protocol-related factors, and for MRI in diffusion sequences, and b-values, should persist as unvariable for reliable measurements to be performed. In the step of protocol optimization, enhanced communication between the different centers can theoretically lead to quality improvement through enhanced patient safety (e.g., radiation dose reduction), contrast optimization, and image quality [46].

Regarding to the “Report” section, this included data on (a) lesion, as if tumor is detectable or not and the presence of indirect signs: pancreatic atrophy, displaced calcifications in patient with chronic calcific pancreatitis, duct-to-parenchyma ratio greater than 0.34, double duct sign, vessel encasement, vessel deformity, superior mesenteric artery (SMA) to superior mesenteric vein (SMV) ratio greater than 1; the lesion size and site such as the lesion structure and vascularity. On (b) arteries, as normal or variant anatomy; atherosclerotic; presence of involvement (less than or equal to 180° tumor contact of the vessel circumference is described as “abutment” and more than 180° tumor contact of the vessel circumference is referred to as “encasement”); distance between celiac trunk and infiltrated hepatic artery >5 mm. On (c) veins, as normal or variant anatomy; thrombosis (neoplastic or not neoplastic); presence of involvement (less than or equal to 180° tumor contact of the vessel circumference is described as “abutment” and more than 180° tumor contact of the vessel circumference is referred to as “encasement”); for superior mesenteric vein, longitudinal extent of infiltration >20 mm and tumor involvement of first jejunal loop.

The presence and degree of contact between the tumor and the peripancreatic vessels is of paramount importance in determining surgical resectability. According to Lu et al. [47], the vascular involvement by the pancreatic tumor is based on the percentage of circumferential surface contact between the tumor and the adjacent vessel, and that more than 180° of tumor–vessel contact is highly specific for tumor unresectability. In addition, irregularity of the vessel contour (including “tear drop” deformity) or change in caliber is also considered a sign of vascular invasion regardless of the degree of contact between tumor and vessel [48]. Furthermore, several additional imaging findings that are pertinent for surgical planning are the presence of tumor or bland venous thrombosis; the extension of tumor contact with the common hepatic artery (CHA) to the level of the origins of right and left hepatic arteries; the extension of tumor contact to first superior mesenteric artery (SMA) branch and to most proximal draining vein into SMV and the arterial variants, and in particular, origin of the right hepatic artery from the SMA. 

In addition, in this section, we included data on biliary ducts, posterior lamina, loco-regional diffusion (including stomach, spleen, treits, Liver, etc.), node stage and for MRI-SR, liver metastases; for CT-SR, metastases stage (including liver, bone, lung, etc.), peritoneal carcinomatosis, as well as the presence of incidental radiological findings, including acute pancreatitis and pulmonary embolism for CT-SR.

The opportunity and benefit of templates to lead the radiologist’s workflow allows the reporting of all essential radiological findings that might be ignored in an FRT through simple distraction. For example, the radiologist should report the arterial variant, especially the presence of a replaced hepatic artery or hepatomesenteric trunk, since the presence of anatomic arterial variants increases the risk for intraoperative vascular injuries and postoperative complications such as hepatic ischemia, biliary anastomotic leak, and pseudoaneurysms. This information may not be provided if the radiological investigation is not performed in a dedicated center and therefore using a checklist and a systematic search pattern may help to prevent such diagnostic errors. In addition, SRs have been shown to enhance clinical impact on tumor staging and surgical planning for pancreatic cancer [49,50]. Brook et al. compared the results of SR versus FRT of CT findings for the staging and assessment of resectability in PDAC patients, showing that surgeons were more confident about tumor resectability using SR [49].

Extensive proposal of SR is an essential goal in order to offer referring physicians and patients the best quality of service, and for researchers the best quality data in the context of big data development. Implementation is complex, requiring mature technology, organizational and interoperability challenges, especially adequate storage of data, and easy and adequate connections with PACS- and post-processing software. Consequently, introduction of SR should be seen as a comprehensive effort, affecting all domains of radiology [51,52,53,54,55,56,57,58,59,60,61,62].

Despite the promising results obtained, this study has some limitations. First, the panelists were only radiologists; therefore, a multidisciplinary approach is lacking. A multidisciplinary validation of SR would be appropriate. Second, the panelists were of the same nationality; the contribution of experts from multiple countries would allow for broader sharing and would increase the consistency of the SR. Finally, this study was not aimed at assessing the impact of the SR on the clinical setting.

## 5. Conclusions

The present templates, based on a multi-round consensus-building Delphi exercise following in-depth discussion between expert radiologists in gastro-enteric and oncological imaging, promoted the use of SR for CT and MRI evaluation in PDCA patients. For both CT and MR pancreas SR, between the first and second round, a major agreement was reached among the 20 panelists highlighted by the increase of Cα correlation coefficient, overall mean score, and sum of scores. This result is due to the awareness of the need to identify the essential features to be reported in a radiological report and, from another point of view, from the idea that today there is a need to integrate clinical and radiological data.

## Figures and Tables

**Table 1:** Single score and sum of scores of panelists for CT staging structured report (I round).

Panelist Number	A1. Anthropometric Data	A2. Personal Assessments	A3. Allergies and Adverse Reactions	B1. Clinical Information	C1. Exam Data	C2. Use of Contrast Agent and Study Protocol	C3. Adverse Events	D1. Primary Lesion	D2. Artery	D3. Vein	D4. Biliary Tract	D5. Posterior Foil	D6. Loco-regional Diffusion	D7. Locoregional Lymphadenopathies	D8. Distant Metastasis	D9. Acute Pancreatitis	D10. Pulmonary Embolism	D11. Accessory Finds	D12. Conclusions	Sum
1	5	5	5	5	5	5	5	5	5	5	5	5	5	5	5	5	5	5	5	95
2	5	5	5	5	5	5	5	5	5	5	5	5	5	5	5	5	5	5	5	95
3	4	4	5	5	5	5	5	5	5	5	5	5	5	5	4	5	5	5	5	92
4	4	4	5	5	5	5	5	5	5	5	5	5	5	5	4	5	5	5	5	92
5	5	5	5	5	5	5	5	5	5	5	5	5	5	5	5	5	5	5	5	95
6	5	5	5	5	5	5	5	5	5	5	5	5	5	5	5	5	5	5	5	95
7	5	4	5	5	5	5	4	5	5	5	5	5	5	5	5	5	5	5	5	93
8	5	4	5	5	5	5	5	5	5	5	5	5	5	5	5	5	5	5	5	94
9	4	4	4	4	4	4	4	4	4	4	4	4	4	4	4	4	4	4	4	76
10	5	5	5	5	5	5	5	5	5	5	5	5	5	5	5	5	5	5	5	95
11	4	4	3	5	5	5	4	5	5	5	4	5	5	5	5	4	4	5	5	87
12	5	5	5	5	5	5	5	5	5	5	5	5	5	5	5	5	5	5	5	95
13	5	5	5	5	5	5	5	5	5	5	5	5	5	5	5	5	5	5	5	95
14	5	5	5	5	5	5	5	5	5	5	5	5	5	5	5	5	5	5	5	95
15	5	5	5	5	5	5	5	5	5	5	5	5	5	5	5	5	5	5	5	95
16	5	4	2	3	2	4	4	5	3	4	4	4	5	5	5	5	4	5	5	78
17	5	5	5	5	5	5	5	5	5	5	5	5	5	5	5	5	5	5	5	95
18	4	4	5	5	5	5	5	5	5	5	5	5	5	5	5	5	5	4	5	92
19	5	5	5	5	5	5	5	5	5	5	5	5	5	5	5	5	5	5	5	95
20	5	5	5	5	5	5	5	5	5	5	5	5	5	5	5	5	5	5	5	95
Mean	4.75	4.60	4.70	4.85	4.80	4.90	4.80	4.95	4.85	4.90	4.85	4.90	4.95	4.95	4.85	4.90	4.85	4.90	4.95	92.20
Std	0.44	0.50	0.80	0.49	0.70	0.31	0.41	0.22	0.49	0.31	0.37	0.31	0.22	0.22	0.37	0.31	0.37	0.31	0.22	5.57

**Table 2:** Single score and sum of scores of panelists for MR staging structured report (I round).

Panelist Number	A1. Anthropometric Data	A2. Personal Assessments	A3. Allergies and Adverse Reactions	B1. Clinical Information	C1. Exam Data	C2. Study Protocol	C3. Contrast Agent	C4. Adverse Events	D1. Primary Lesion	D2. Artery	D3. Vein	D4. Biliary Tract	D5. Posterior Foil	D6. Loco-regional Diffusion	D7. Locoregional Lymphadenopathies	D8. Distant Metastasis	D9. Acute Pancreatitis	D11. Accessory Finds	D12. Conclusions	Sum
1	5	4	5	5	5	5	5	5	5	5	5	5	5	5	5	5	5	5	5	94
2	5	5	5	5	5	5	5	5	5	5	5	5	5	5	5	5	5	5	5	95
3	4	4	4	4	5	5	5	5	5	5	4	5	5	5	5	5	5	5	4	89
4	4	4	5	5	5	5	5	5	5	5	5	5	5	5	5	5	5	5	5	93
5	4	4	5	5	5	5	5	5	5	5	5	5	5	5	5	5	5	5	5	93
6	4	4	4	4	4	4	4	4	4	4	4	4	4	4	4	4	4	4	4	76
7	4	4	4	5	5	5	5	5	5	5	5	5	5	5	5	5	5	4	5	91
8	4	2	2	3	3	2	3	4	4	5	5	4	5	5	5	5	4	5	5	75
9	5	4	5	5	5	5	5	5	5	5	5	5	5	5	5	5	5	5	5	94
10	5	4	5	5	5	5	5	4	5	5	5	5	5	5	5	5	5	5	5	93
11	5	3	5	5	3	1	5	5	5	5	5	5	5	5	5	5	5	5	5	87
12	5	4	5	5	5	5	5	5	5	5	5	5	5	5	5	5	5	5	5	94
13	4	4	4	4	4	4	4	4	4	4	4	4	4	4	4	4	4	4	4	76
14	5	5	5	5	5	5	5	5	5	5	5	5	5	5	5	5	5	5	5	95
15	4	3	3	4	5	5	5	4	5	5	5	5	5	5	5	5	4	4	5	86
16	5	5	5	5	5	5	5	5	5	5	5	5	5	5	5	5	5	5	5	95
17	4	4	5	5	5	5	5	5	5	5	5	5	5	5	5	5	5	5	5	93
18	4	4	5	5	5	5	5	5	5	5	5	5	5	5	5	5	5	5	5	93
19	5	5	5	5	5	5	5	5	5	5	5	5	5	5	5	5	5	5	5	95
20	4	4	4	5	5	5	5	5	5	5	5	5	5	5	5	5	5	4	5	91
Mean	4.45	4.00	4.50	4.70	4.70	4.55	4.80	4.75	4.85	4.90	4.85	4.85	4.90	4.90	4.90	4.90	4.80	4.75	4.85	89.90
Std	0.51	0.73	0.83	0.57	0.66	1.10	0.52	0.44	0.37	0.31	0.37	0.37	0.31	0.31	0.31	0.31	0.41	0.44	0.37	6.64

**Table 3:** Single score and sum of scores of panelists for CT staging structured report (II round).

Panelist Number	A1. Anthropometric Data	A2. Personal Assessments	A3. Allergies and Adverse Reactions	B1. Clinical Information	C1. Exam Data	C2. Use of Contrast Agent and Study Protocol	C3. Adverse Events	D1. Primary Lesion	D2. Artery	D3. Vein	D4. Biliary Tract	D5. Posterior Foil	D6. Loco-regional Diffusion	D7. Locoregional Lymphadenopathies	D8. Distant Metastasis	D9. Acute Pancreatitis	D10. Pulmonary Embolism	D11. Accessory Finds	D12. Conclusions	Sum
1	5	5	5	5	5	5	5	5	5	5	5	5	5	5	5	5	5	5	5	95
2	5	5	5	5	5	5	5	5	5	5	5	5	5	5	5	5	5	5	5	95
3	4	4	3	5	5	5	3	5	5	5	4	5	5	5	5	4	4	4	5	85
4	5	5	5	5	5	5	5	5	5	5	5	5	5	5	5	5	5	5	5	95
5	5	5	5	5	5	5	5	5	5	5	5	5	5	5	5	5	5	5	5	95
6	5	5	5	5	5	5	5	5	5	5	5	5	5	5	5	3	4	5	5	92
7	5	4	5	3	5	4	5	3	4	4	4	5	5	5	5	4	5	5	5	85
8	5	5	5	5	5	5	5	5	5	5	5	5	5	5	5	5	5	5	5	95
9	4	4	5	5	5	5	5	5	5	5	5	5	5	5	5	5	5	4	5	92
10	5	5	5	5	5	5	5	5	5	5	5	5	5	5	5	5	5	5	5	95
11	5	5	5	5	5	5	5	5	5	5	5	5	5	5	5	5	5	5	5	95
12	5	5	5	5	5	5	5	5	5	5	5	5	5	5	5	5	5	5	5	95
13	5	5	5	5	5	5	5	5	5	5	5	5	5	5	5	5	5	5	5	95
14	5	5	5	5	5	5	5	5	5	5	5	5	5	5	5	5	5	5	5	95
15	4	4	3	5	5	5	3	5	5	5	4	5	5	5	5	4	4	4	5	85
16	5	5	5	5	5	5	5	5	5	5	5	5	5	5	5	5	5	5	5	95
17	5	5	5	5	5	5	5	5	5	5	5	5	5	5	5	5	5	5	5	95
18	5	5	5	5	4	5	5	5	5	5	5	5	5	5	5	3	4	5	5	91
19	5	4	5	3	5	4	5	3	4	4	4	5	5	5	5	4	5	5	5	85
20	5	5	5	5	5	5	5	5	5	5	5	5	5	5	5	5	5	5	5	95
Mean	4.85	4.75	4.80	4.80	4.95	4.90	4.80	4.80	4.90	4.90	4.80	5.00	5.00	5.00	5.00	4.60	4.80	4.85	5.00	92.50
Std	0.37	0.44	0.62	0.62	0.22	0.31	0.62	0.62	0.31	0.31	0.41	0.00	0.00	0.00	0.00	0.68	0.41	0.37	0.00	4.03

**Table 4:** Single score and sum of scores of panelists for MR staging structured report (II round).

Panelist Number	A1. Anthropometric Data	A2. Personal Assessments	A3. Allergies and Adverse reactions	B1. Clinical Information	C1. Exam Data	C2. Use of Contrast Agent and Study Protocol	C3. Adverse Events	D1. Primary Lesion	D2. Artery	D3. Vein	D4. Biliary Tract	D5. Posterior Foil	D6. Loco-regional Diffusion	D7. Locoregional Lymphadenopathies	D8. Distant Metastasis	D9. Acute Pancreatitis	D10. Pulmonary Embolism	D11. Accessory Finds	D12. Conclusions	Sum
1	5	5	5	5	5	5	5	5	5	5	5	5	5	5	5	5	5	5	5	95
2	5	5	5	5	5	5	5	5	5	5	5	5	5	5	5	5	5	5	5	95
3	5	3	3	5	5	5	5	3	5	5	5	4	5	5	4	5	4	4	5	85
4	5	5	5	5	5	5	5	5	5	5	5	5	5	5	5	5	5	5	5	95
5	5	5	5	5	5	5	5	5	5	5	5	5	5	5	5	5	5	5	5	95
6	5	5	5	5	5	5	5	5	5	5	5	5	5	5	5	5	5	5	5	95
7	5	4	5	5	5	4	4	5	5	5	4	4	5	5	5	5	5	5	5	90
8	5	5	5	5	5	5	5	5	5	5	5	5	5	5	5	5	5	5	5	95
9	4	4	5	5	5	5	5	5	5	5	5	5	5	5	5	5	5	5	5	93
10	5	5	5	5	5	5	5	5	5	5	5	5	5	5	5	5	5	5	5	95
11	5	5	5	5	5	5	5	5	5	5	5	5	5	5	5	5	5	5	5	95
12	5	5	5	5	5	5	5	5	5	5	5	5	5	5	5	5	5	5	5	95
13	5	5	5	5	5	5	5	5	5	5	5	5	5	5	5	5	5	5	5	95
14	5	5	5	5	5	5	5	5	5	5	5	5	5	5	5	5	5	5	5	95
15	3	3	3	5	5	5	5	3	5	5	5	5	5	5	5	5	4	5	5	86
16	5	5	5	5	5	5	5	5	5	5	5	5	5	5	5	5	5	5	5	95
17	5	5	5	5	5	5	5	5	5	5	5	5	5	5	5	5	5	5	5	95
18	5	5	5	5	5	5	5	5	5	5	5	5	5	5	5	5	5	5	5	95
19	5	5	5	3	5	4	5	5	3	4	5	5	5	5	5	5	4	5	5	88
20	5	5	5	5	5	5	5	5	5	5	5	5	5	5	5	5	5	5	5	95
Mean	4.85	4.70	4.80	4.90	5.00	4.90	4.95	4.80	4.90	4.95	4.95	4.90	5.00	5.00	4.95	5.00	4.85	4.95	5.00	93.35
Std	0.49	0.66	0.62	0.45	0.00	0.31	0.22	0.62	0.45	0.22	0.22	0.31	0.00	0.00	0.22	0.00	0.37	0.22	0.00	3.28

## Data Availability

All data are reported in the manuscript.

## Supplementary Materials

The following are available online at https://www.mdpi.com/article/10.3390/diagnostics11112033/s1.
