# Supplementary material for: Structured Reporting of Computed Tomography and Magnetic Resonance in the Staging of Pancreatic Adenocarcinoma: A Delphi Consensus Proposal
Source: Diagnostics (Basel). 2021 Nov 3;11(11):2033. doi: 10.3390/diagnostics11112033 (PMC8621603; doi:10.3390/diagnostics11112033)
Supplement: Supplementary file 1 [file diagnostics-11-02033-s001.zip › diagnostics-1423817-supplementary.pdf]

diagnostics

MDPI

Section S1

PATIENT CLINICAL DATA *(imported from RIS)*

FIELD	DETAIL	ADMITTED VALUES
ANTHROPOMETRIC DATA		
Weight	(kg) *[Numeric]*	
Height	(cm) *[Numeric]*	
BMI	*[Numeric]* (automatically calculated)	
BSA	*[Numeric]* (automatically calculated)	
Age	(years) *[Numeric]*	
Age range<br>(filled in automatically)	• < 50 years<br>• > 50 years	
PATIENT HISTORY		
Family history of pancreatic cancer<br>(visible only if "Yes" and repeatable)	Yes/No	
	Degree of kinship	• Mother<br>• Father<br>• Brother(s)/sister(s)

		• Maternal grandparent(s)<br>• Paternal grandparent(s)<br>• Uncle(s)/aunt(s)<br>• Other *[Free text]*
	Notes	*[Free text]*
Personal history of other cancers	Yes/No	
	Notes	*[Free text]*
Predisposing diseases<br>(visible only if "Yes" and repeatable)	Yes/No	
	Type	• Obesity<br>• Diabetes mellitus<br>• Chronic pancreatitis<br>• Prior gastrectomy
	Notes	*[Free text]*
Hereditary syndromes<br>(visible only if "Yes" and repeatable)	Type	• Peutz-Jeghers syndrome<br>• familial atypical multiple mole melanoma (FAMMM) syndrome<br>• hereditary pancreatitis<br>• autoimmune syndromes (pancreatitis, etc.)

		• Lynch syndrome<br>• hereditary nonpolyposis colorectal cancer (HNPCC)<br>• familial breast cancer<br>• ataxia-telangiectasia<br>• other *[Free text]*	
Other genetic mutations	Yes/No	• BRCA-2<br>• other *[Free text]*	
Lifestyle/dietary habits	Smoke	Yes/No	
(visible only if “Yes” and repeatable)	SMOKING DETAILS (visible only if Smoke = “Yes”)		
		Smoker	• Current smoker<br>• Former smoker
		Cigarette smoke	Yes/No
		Number of daily cigarettes (if “Current smoker”)	*[Numeric]*
		Smoking years	*[Numeric]*
		Years from cessation (if “Former smoker”)	• ≤15<br>• >15

		Cigarettes per year (pack-year)<br>(if "Former smoker" or "Current smoker")	*[Numeric]* (automatically calculated)*<br><br>*(Number of daily cigarettes x smoking years / 20)
		Vaping	Yes/No
		Number of daily electronic cigarettes refills<br>(if vaping = "Yes")	*[Numeric]*
		Number of years<br>(if vaping = "Yes")	*[Numeric]*
		Notes	*[Free text]*
	High alcohol intake	Yes (more than 1 glass/day for women and 2 glasses/day for men)<br><br>No	
	High meat intake	Yes (white or red meat intake more than 3 times/week)<br><br>No	
	High cured meat intake	Yes (cured meat intake more than once a week)<br><br>No	
	Low vegetable intake	Yes (less than 2 servings/day)<br><br>No	

	Low fruit intake	Yes (less than 3 whole fruits/day)<br><br>No
	Notes	*[Free text]*
ALLERGIES AND ADVERSE REACTIONS		
Reported allergies<br><br>(visible only if "Yes" and repeatable)	Yes/No	
	Type	• Drug-related (n of drugs)<br>• Contrast medium-related (n of contrast media)<br>• Drug-unrelated
	Active principle/molecule<br>[if drug- or contrast medium-related allergy]	*[Free text]*
	Commercial name<br><br>[if drug- or contrast medium-related allergy]	*[Free text]*
	Notes	*[Free text]*
PREVIOUS adverse reactions<br><br>(visible only if "Yes" and repeatable)	Yes/No	
	Date	month/year *[mm/yyyy]*
	Type	• Contrast medium-related / unrelated

	Degree	• Mild<br>• Moderate<br>• Severe
	Time of onset	• Early<br>• Late
	Notes	*[Free text]*
Antiallergic premedication	Yes/No	
	Treatment	• Steroid<br>• Antihistamine
	Complete	Yes/No
	Notes	*[Free text]*
Nephroprotective protocol	Yes/No	
	Complete	Yes/No
	Serum creatinine	*[Numeric]* (mg/dl)
	GFR (Glomerular Filtration Rate)	*[Numeric]* (ml/min)<br><br>https://www.merckmanuals.com/medical-calculators/GFR_CKD_EPI-it.htm (sex, race, age, serum creatinine)

	Notes	*[Free text]*

CLINICAL EVALUATION

FIELD	DETAIL	ADMITTED VALUES
CLINICAL INFORMATION	Yes/No	
Prior imaging tests<br><br>(visible only if "Yes" and repeatable)	Type	• Gastroscopy<br>• Endoscopic ultrasonography<br>• CT<br>• MRI<br>• Ultrasonography<br>• CEUS<br>• ^{18}F-FDG PET-CT<br>• ^{68}Ga-SA PET-CT<br>• Other *[Free text]*
	Date	*[dd/mm/yyyy]*
	Notes	*[Free text]*
Histopathological examination on biopsy specimen	Yes/No	
Histologic grade	• Gx	

	• G1<br>• G2<br>• G3
Histologic type	• Ductal adenocarcinoma<br>• Adenosquamous carcinoma<br>• Hepatoid carcinoma<br>• Medullary carcinoma<br>• Mucinous noncystic carcinoma (colloid carcinoma)<br>• Signet ring cell carcinoma<br>• Undifferentiated carcinoma<br>• Undifferentiated carcinoma with osteoclast-like giant cells<br>• Acinar cell carcinoma<br>• Acinar cell cystadenocarcinoma<br>• Intraductal papillary mucinous neoplasm with associated invasive carcinoma<br>• Intraductal tubulopapillary neoplasm with associated invasive carcinoma<br>• Mucinous cystic neoplasm with associated invasive carcinoma<br>• Pancreatoblastoma<br>• Serous cystadenocarcinoma

	• Solid pseudopapillary neoplasm<br>• Neuroendocrine carcinoma<br>• Small cell neuroendocrine carcinoma<br>• Large cell neuroendocrine carcinoma<br>• Mixed acinar ductal carcinoma<br>• Mixed acinar neuroendocrine carcinoma<br>• Mixed acinar neuroendocrine ductal carcinoma<br>• Mixed neuroendocrine ductal carcinoma	
Ca19.9 level	*[Numeric]*	
CEA level	*[Numeric]*	
Blood count	*[Numeric]*	
Serum creatinine	*[Numeric]*	
Liver function	• Normal<br>• Impaired	
Symptoms	Yes/No	If yes:<br>• pain (yes/no)<br>• jaundice (yes/no)<br>• diarrhea (yes/no)<br>• other *[Free text]*

IMAGING PROTOCOL

FIELD	DETAIL	ADMITTED VALUES
IMAGING DATA		
Date of examination	Date *[dd/mm/yyyy]*	
Clinical indication	Primary staging	
Scanner brand and model	*[Free text]*	
CT protocol	Number of detector rows	*[Numeric]*
	Precontrast scan<br><br>***(*details visible only if "Yes")***	*Yes/No<br><br>Dual energy (Yes/No)<br><br>Slice thickness (mm) *[Numeric]*<br><br>Convolution kernel(s) *[Free text]*<br><br>Body area *[multiple choice]*:<br>• abdomen<br>• chest<br>• neck<br>• brain
	Post-contrast scan	**[Numeric]*

	(*details repeatable for each post-contrast scan)	Post-contrast phase(s) *[multiple choice]*:<br>• early arterial<br>• pancreatic<br>• venous<br>• late<br>Dual energy (Yes/No)<br>Slice thickness (mm) *[Numeric]*<br>Convolution kernel(s) *[Free text]*<br>Body area *[multiple choice]*:<br>• abdomen<br>• chest<br>• neck<br>• brain
Radiation exposure	Class of radiation exposure	*[Numeric]*
CONTRAST MEDIUM		
Use of contrast medium	Yes/No	
(visible only if "Yes")	Active principle	• Iobitridol

		• Iodixanol<br>• Iohexol<br>• Iomeprol<br>• Iopromide<br>• Ioversol
	Commercial name	*[Free text]*
	Volume	*[Numeric]* (ml)
	Flow rate	*[Numeric]* (ml/sec)
	Iodine concentration	*[Numeric]* (mg I/ml)
ADVERSE EVENTS		
ONGOING adverse events<br><br>(visible only if "Yes")	Yes/No	
	Date and hour of event	*[dd/mm/yyyy, hour]*
	Degree	• Mild<br>• Moderate<br>• Severe
	Time of onset	• Early<br>• Late<br><br>Minutes *[Numeric]* (optional)

	Type	ALLERGIC / ALLERGIC-LIKE<br>**Mild**<br>• Sparse wheals/itch<br>• Skin edema<br>• Mild itching / feeling like ”velvet in the throat“<br>• Nasal congestion<br>• Sneezing<br>• Conjunctivitis<br>• Rhinorrhea<br><br>**Moderate**<br>• Diffuse wheals/intense itch<br>• Diffuse skin edema<br>• Facial edema without dyspnea<br>• Feeling of choking or hoarseness<br>• Wheezing / mild bronchospasm without hypoxia<br><br>**Severe**<br>• Dyspnea

		• Erythema – diffuse mucocutaneous symptoms<br>• Laryngeal edema with stridor and/or hypoxia<br>• Wheezing / bronchospasm<br>• Significant hypoxia<br>• Anaphylactic shock (severe hypotension and brady-tachyarrhythmia)<br><br>NON-ALLERGIC<br>**Mild**<br>• Mild nausea/limited vomiting<br>• Transient chills / heat / redness<br>• Headache / dizziness / anxiety / altered taste<br>• Slight increase in blood pressure<br>• Self-limiting vasovagal reaction<br>**Moderate**<br>• Prolonged nausea/vomiting<br>• Elevated arterial blood pressure<br>• Isolated chest pain

		• Vasovagal reaction<br><br>**Severe**<br>• Treatment-refractory vasovagal reaction<br>• Arrhythmia<br>• Convulsions<br>• Severe arterial hypertension<br><br>CONTRAST MEDIUM EXTRAVASATION
	Type of treatment	• Wait and see<br>• Drug therapy (specify in "Notes" field)<br>• Anesthesiologist's intervention required
	Event resolution	• Spontaneous<br>• After treatment<br>• After hospitalization<br>• Other [*Free text]*

REPORT

FIELD	DETAIL	ADMITTED VALUES
DIAGNOSIS		
PRIMARY TUMOR		
Lesion	Detectable (yes/no)	If no:<br>Indirect signs:<br>• pancreatic atrophy<br>• displaced calcifications in patient with chronic calcific pancreatitis<br>• duct-to-parenchyma ratio greater than 0.34<br>• double duct sign<br>• vessel encasement<br>• vessel deformity<br>• superior mesenteric artery (SMA) to superior mesenteric vein (SMV) ratio greater than 1<br><br>If yes: Size (mm *[Numeric]*)

	Structure	• Solid<br>• Cystic<br>• Mixed (solid/cystic)<br><br>• Description *[Free text]*
	Site	• Head, with option to specify:<br>• paraduodenal portion (i.e., right of gastroduodenal artery)<br>• paravascular portion (i.e., left of gastroduodenal artery)<br>• upper portion<br>• lower portion<br>• Uncinate process<br>• Isthmus<br>• Body<br>• Tail
	Vascularity	• Hypovascular<br>• Isovascular<br>• Hypervascular<br>• Delayed enhancement

Arteries	Anatomy	• Normal<br>• Variant (*[Free text]*)
	Superior mesenteric artery<br>*(<u>note</u>: vessels are considered to be not infiltrated when they are surrounded by a fat ring)*	• Not infiltrated<br>• <180°<br>• >180°<br>• Atherosclerotic
	Celiac trunk<br>*(<u>note</u>: vessels are considered to be not infiltrated when they are surrounded by a fat ring)*	• Not infiltrated<br>• <180°<br>• >180°<br>• Atherosclerotic
	Hepatic artery<br>*(<u>note</u>: vessels are considered to be not infiltrated when they are surrounded by a fat ring)*	• Not infiltrated<br>• <180°<br>• >180°<br>• Atherosclerotic
	*Distance between celiac trunk and infiltrated hepatic artery >5mm	Yes/No
Veins	Anatomy	• Normal<br>• Variant (*[Free text]*)

	Superior mesenteric vein<br>(*<u>note</u>: vessels are considered to be not infiltrated when they are surrounded by a fat ring)*	• Not infiltrated<br>• <180°<br>• >180°<br><br>• Thrombosis (neoplastic / non-neoplastic)<br>• Longitudinal extent of infiltration >20mm (yes/no)<br>• Tumor involves first jejunal loop (yes/no)
	Portal vein<br>(*<u>note</u>: vessels are considered to be not infiltrated when they are surrounded by a fat ring)*	• Not infiltrated<br>• <180°<br>• >180°<br><br>• Thrombosis (neoplastic / non-neoplastic)
	Splenic vein<br>(*<u>note</u>: vessels are considered to be not infiltrated when they are surrounded by a fat ring)*	• Not infiltrated<br>• <180°<br>• >180°<br><br>• Thrombosis (neoplastic / non-neoplastic)
Biliary ducts	Dilated	Yes/No; if yes:

		• diameter of left-sided ducts (mm) *[Numeric]*<br>• diameter of right-sided ducts (mm) *[Numeric]*<br>• diameter of common bile duct (mm) *[Numeric]*
Posterior lamina	Infiltration	• Yes<br>• No<br>• Uncertain (*[Free text]*)
Loco-regional diffusion	Stomach	Infiltration (yes/no)
	Spleen	Infiltration (yes/no)
	Duodenum	Infiltration (yes/no)
	Treitz	Infiltration (yes/no)
	Common bile duct	Infiltration (yes/no)
	Liver	Infiltration (yes/no)
	Other	*[Free text]*
Notes	*[Free text]*	
Peritoneal effusion	Peripancreatic effusion	Yes/No
	Supramesocolic effusion	Yes/No

	Pelvic effusion	Yes/No
	Supra- and submesocolic effusion	Yes/No
Peritoneal carcinomatosis	• No<br>• Yes (description *[Free text]*)	
Omentum	Infiltration	• Yes<br>• No<br>• Uncertain (*[Free text]*)

LOCO-REGIONAL LYMPHOADENOP-ATHIES	Yes/No; if yes:<br>• Peripancreatic<br>• Celiac<br>• Para-aortic<br>*(note: lymph nodes with long axis >5mm should be reported)*
DISTANT METASTASES	Yes/No
Liver	Yes/No; if yes:<br>Number of detectable lesions *[Numeric]*<br>For each target lesion (up to 2):<br>• site *[Liver segment]*

	• maximum diameter on axial images (mm) *[Numeric]*<br>• diameter perpendicular to maximum diameter (mm) *[Numeric]*<br>• structure *[Free text]*
Lung	Yes/No; if yes:<br>Number of detectable lesions *[Numeric]*<br>For each target lesion (up to 2):<br>• site *[Lung lobe, Lung segment]*<br>• maximum diameter on axial images (mm) *[Numeric]*<br>• diameter perpendicular to maximum diameter (mm) *[Numeric]*<br>Lymphangitic carcinomatosis (yes/no; if yes, site *[Free text]*)
Non-regional lymph nodes	Yes/No; if yes:<br>• site *[Free text]*<br>• maximum diameter on axial images (mm) *[Numeric]*<br>• diameter perpendicular to maximum diameter (mm) *[Numeric]*
Other organs (incl. bone)	• yes (site and type *[Free text]*)<br>• no

Acute pancreatitis	• No<br>• Yes, ongoing (report findings *[Free text]*)<br>• Yes, sequelae (pseudocysts *[Yes/no]*)
Pulmonary embolism	• No<br>• Yes (site and extent *[Free text]*)

INCIDENTAL FINDINGS	
Brain	*[Free text]*
Neck	*[Free text]*
Chest	*[Free text]*
Abdomen	*[Free text]*

CONCLUSIONS AND RECOMMENDATIONS	*[Free text]*

IMAGES

FIELD	DETAIL	ADMITTED VALUES

Key images	Key images	*[Images]*

Section S2

PATIENT CLINICAL DATA *(imported from RIS)*

FIELD	DETAIL	ADMITTED VALUES
ANTHROPOMETRIC DATA		
Weight	(kg) *[Numeric]*	
Height	(cm) *[Numeric]*	
BMI	*[Numeric]* (automatically calculated)	
BSA	*[Numeric]* (automatically calculated)	
Age	(years) *[Numeric]*	
Age range<br>(filled in automatically)	• < 50 years<br>• > 50 years	
PATIENT HISTORY		
Family history of pancreatic cancer<br>(visible only if "Yes" and repeatable)	Yes/No	
	Degree of kinship	• Mother<br>• Father<br>• Brother(s)/sister(s)<br>• Maternal grandparent(s)<br>• Paternal grandparent(s)

		• Uncle(s)/aunt(s)<br>• Other *[Free text]*
	Notes	*[Free text]*
Personal history of other cancers	Yes/No	
	Notes	*[Free text]*
Predisposing diseases<br>(visible only if "Yes" and repeatable)	Yes/No	
	Type	• Obesity<br>• Diabetes mellitus<br>• Chronic pancreatitis<br>• Prior gastrectomy
	Notes	*[Free text]*
Hereditary syndromes<br>(visible only if "Yes" and repeatable)	Type	• Peutz-Jeghers syndrome<br>• familial atypical multiple mole melanoma (FAMMM) syndrome<br>• hereditary pancreatitis<br>• autoimmune syndromes (pancreatitis, etc.)<br>• Lynch syndrome

		• hereditary nonpolyposis colorectal cancer (HNPCC)<br>• familial breast cancer<br>• ataxia-telangiectasia<br>• other *[Free text]*	
Other genetic mutations	Yes/No	• BRCA-2<br>• other *[Free text]*	
Lifestyle/dietary habits<br><br>(visible only if "Yes" and repeatable)	Smoke	Yes/No	
	SMOKING DETAILS (visible only if Smoke = "Yes")		
		Smoker	• Current smoker<br>• Former smoker
		Cigarette smoke	Yes/No
		Number of daily cigarettes (if "Current smoker")	*[Numeric]*
		Smoking years	*[Numeric]*
		Years from cessation (if "Former smoker")	• ≤15<br>• >15
		Cigarettes per year (pack-year)	*[Numeric]* (automatically calculated)*

		(if "Former smoker" or "Current smoker")	*(Number of daily cigarettes x smoking years / 20)
		Vaping	Yes/No
		Number of daily electronic cigarettes refills (if vaping = "Yes")	*[Numeric]*
		Number of years (if vaping = "Yes")	*[Numeric]*
		Notes	*[Free text]*
	High alcohol intake	Yes (more than 1 glass/day for women and 2 glasses/day for men)<br>No	
	High meat intake	Yes (white or red meat intake more than 3 times/week)<br>No	
	High cured meat intake	Yes (cured meat intake more than once a week)<br>No	
	Low vegetable intake	Yes (less than 2 servings/day)<br>No	
	Low fruit intake	Yes (less than 3 whole fruits/day)<br>No	
	Notes	*[Free text]*	

ALLERGIES AND ADVERSE REACTIONS		
Reported allergies<br><br>(visible only if "Yes" and repeatable)	Yes/No	
	Type	• Drug-related (n of drugs)<br>• Contrast medium-related (n of contrast media)<br>• Drug-unrelated
	Active principle/molecule<br>[if drug- or contrast medium-related allergy]	*[Free text]*
	Commercial name<br><br>[if drug- or contrast medium-related allergy]	*[Free text]*
	Notes	*[Free text]*
PREVIOUS adverse reactions<br><br>(visible only if "Yes" and repeatable)	Yes/No	
	Date	month/year *[mm/yyyy]*
	Type	• Contrast medium-related / unrelated
	Degree	• Mild<br>• Moderate<br>• Severe

	Time of onset	• Early<br>• Late
	Notes	*[Free text]*
Antiallergic premedication	Yes/No	
	Treatment	• Steroid<br>• Antihistamine
	Complete	Yes/No
	Notes	*[Free text]*
Nephroprotective protocol	Yes/No	
	Complete	Yes/No
	Serum creatinine	*[Numeric]* (mg/dl)
	GFR (Glomerular Filtration Rate)	*[Numeric]* (ml/min)<br><br>https://www.merckmanuals.com/medical-calculators/GFR_CKD_EPI-it.htm (sex, race, age, serum creatinine)
	Notes	*[Free text]*

CLINICAL EVALUATION

FIELD	DETAIL	ADMITTED VALUES
CLINICAL INFORMATION	Yes/No	
Prior imaging tests<br>(visible only if "Yes" and repeatable)	Type	• Gastroscopy<br>• Endoscopic ultrasonography<br>• CT<br>• MRI<br>• Ultrasonography<br>• CEUS<br>• ^{18}F-FDG PET-CT<br>• ^{68}Ga-SA PET-CT<br>• Other *[Free text]*
	Date	*[dd/mm/yyyy]*
	Notes	*[Free text]*
Histopathological examination on biopsy specimen	Yes/No	
Histologic grade	• Gx<br>• G1	

	• G2<br>• G3
Histologic type	• Ductal adenocarcinoma<br>• Adenosquamous carcinoma<br>• Hepatoid carcinoma<br>• Medullary carcinoma<br>• Mucinous noncystic carcinoma (colloid carcinoma)<br>• Signet ring cell carcinoma<br>• Undifferentiated carcinoma<br>• Undifferentiated carcinoma with osteoclast-like giant cells<br>• Acinar cell carcinoma<br>• Acinar cell cystadenocarcinoma<br>• Intraductal papillary mucinous neoplasm with associated invasive carcinoma<br>• Intraductal tubulopapillary neoplasm with associated invasive carcinoma<br>• Mucinous cystic neoplasm with associated invasive carcinoma<br>• Pancreatoblastoma<br>• Serous cystadenocarcinoma<br>• Solid pseudopapillary neoplasm

	• Neuroendocrine carcinoma<br>• Small cell neuroendocrine carcinoma<br>• Large cell neuroendocrine carcinoma<br>• Mixed acinar ductal carcinoma<br>• Mixed acinar neuroendocrine carcinoma<br>• Mixed acinar neuroendocrine ductal carcinoma<br>• Mixed neuroendocrine ductal carcinoma	
Ca19.9 level	*[Numeric]*	
CEA level	*[Numeric]*	
Blood count	*[Numeric]*	
Serum creatinine	*[Numeric]*	
Liver function	• Normal<br>• Impaired	
Symptoms	Yes/No	If yes:<br>• pain (yes/no)<br>• jaundice (yes/no)<br>• diarrhea (yes/no)<br>• other *[Free text]*

IMAGING PROTOCOL

FIELD	DETAIL	ADMITTED VALUES
IMAGING DATA		
Date of examination	Date *[dd/mm/yyyy]*	
Clinical indication	Primary staging	
Scanner brand and model	*[Free text]*	
CT protocol	Number of detector rows	*[Numeric]*
	Precontrast scan<br>***(*details visible only if "Yes")***	*Yes/No<br>Dual energy (Yes/No)<br>Slice thickness (mm) *[Numeric]*<br>Convolution kernel(s) *[Free text]*<br>Body area *[multiple choice]*:<br>• abdomen<br>• chest<br>• neck<br>• brain
	Post-contrast scan	**[Numeric]*

	(*details repeatable for each post-contrast scan)	Post-contrast phase(s) *[multiple choice]*:<br>• early arterial<br>• pancreatic<br>• venous<br>• late<br>Dual energy (Yes/No)<br>Slice thickness (mm) *[Numeric]*<br>Convolution kernel(s) *[Free text]*<br>Body area *[multiple choice]*:<br>• abdomen<br>• chest<br>• neck<br>• brain
Radiation exposure	Class of radiation exposure	*[Numeric]*
CONTRAST MEDIUM		
Use of contrast medium	Yes/No	
(visible only if "Yes")	Active principle	• Iobitridol

		• Iodixanol<br>• Iohexol<br>• Iomeprol<br>• Iopromide<br>• Ioversol
	Commercial name	*[Free text]*
	Volume	*[Numeric]* (ml)
	Flow rate	*[Numeric]* (ml/sec)
	Iodine concentration	*[Numeric]* (mg I/ml)
ADVERSE EVENTS		
ONGOING adverse events<br><br>(visible only if "Yes")	Yes/No	
	Date and hour of event	*[dd/mm/yyyy, hour]*
	Degree	• Mild<br>• Moderate<br>• Severe
	Time of onset	• Early<br>• Late<br><br>Minutes *[Numeric]* (optional)

	Type	ALLERGIC / ALLERGIC-LIKE<br>**Mild**<br>• Sparse wheals/itch<br>• Skin edema<br>• Mild itching / feeling like ”velvet in the throat“<br>• Nasal congestion<br>• Sneezing<br>• Conjunctivitis<br>• Rhinorrhea<br><br>**Moderate**<br>• Diffuse wheals/intense itch<br>• Diffuse skin edema<br>• Facial edema without dyspnea<br>• Feeling of choking or hoarseness<br>• Wheezing / mild bronchospasm without hypoxia<br><br>**Severe**<br>• Dyspnea

		• Erythema – diffuse mucocutaneous symptoms<br>• Laryngeal edema with stridor and/or hypoxia<br>• Wheezing / bronchospasm<br>• Significant hypoxia<br>• Anaphylactic shock (severe hypotension and brady-tachyarrhythmia)<br><br>NON-ALLERGIC<br>**Mild**<br>• Mild nausea/limited vomiting<br>• Transient chills / heat / redness<br>• Headache / dizziness / anxiety / altered taste<br>• Slight increase in blood pressure<br>• Self-limiting vasovagal reaction<br>**Moderate**<br>• Prolonged nausea/vomiting<br>• Elevated arterial blood pressure<br>• Isolated chest pain

		• Vasovagal reaction<br><br>**Severe**<br>• Treatment-refractory vasovagal reaction<br>• Arrhythmia<br>• Convulsions<br>• Severe arterial hypertension<br><br>CONTRAST MEDIUM EXTRAVASATION
	Type of treatment	• Wait and see<br>• Drug therapy (specify in "Notes" field)<br>• Anesthesiologist's intervention required
	Event resolution	• Spontaneous<br>• After treatment<br>• After hospitalization<br>• Other [*Free text]*

REPORT

FIELD	DETAIL	ADMITTED VALUES
DIAGNOSIS		
PRIMARY TUMOR		
Lesion	Detectable (yes/no)	If no:<br>Indirect signs:<br>• pancreatic atrophy<br>• displaced calcifications in patient with chronic calcific pancreatitis<br>• duct-to-parenchyma ratio greater than 0.34<br>• double duct sign<br>• vessel encasement<br>• vessel deformity<br>• superior mesenteric artery (SMA) to superior mesenteric vein (SMV) ratio greater than 1<br><br>If yes: Size (mm *[Numeric]*)

	Structure	• Solid<br>• Cystic<br>• Mixed (solid/cystic)<br><br>• Description *[Free text]*
	Site	• Head, with option to specify:<br>• paraduodenal portion (i.e., right of gastroduodenal artery)<br>• paravascular portion (i.e., left of gastroduodenal artery)<br>• upper portion<br>• lower portion<br>• Uncinate process<br>• Isthmus<br>• Body<br>• Tail
	Vascularity	• Hypovascular<br>• Isovascular<br>• Hypervascular<br>• Delayed enhancement

Arteries	Anatomy	• Normal<br>• Variant (*[Free text]*)
	Superior mesenteric artery<br>*(<u>note</u>: vessels are considered to be not infiltrated when they are surrounded by a fat ring)*	• Not infiltrated<br>• <180°<br>• >180°<br>• Atherosclerotic
	Celiac trunk<br>*(<u>note</u>: vessels are considered to be not infiltrated when they are surrounded by a fat ring)*	• Not infiltrated<br>• <180°<br>• >180°<br>• Atherosclerotic
	Hepatic artery<br>*(<u>note</u>: vessels are considered to be not infiltrated when they are surrounded by a fat ring)*	• Not infiltrated<br>• <180°<br>• >180°<br>• Atherosclerotic
	*Distance between celiac trunk and infiltrated hepatic artery >5mm	Yes/No
Veins	Anatomy	• Normal<br>• Variant (*[Free text]*)

	Superior mesenteric vein<br><br>*(<u>note</u>: vessels are considered to be not infiltrated when they are surrounded by a fat ring)*	• Not infiltrated<br>• <180°<br>• >180°<br><br>• Thrombosis (neoplastic / non-neoplastic)<br>• Longitudinal extent of infiltration >20mm (yes/no)<br>• Tumor involves first jejunal loop (yes/no)
	Portal vein<br><br>*(<u>note</u>: vessels are considered to be not infiltrated when they are surrounded by a fat ring)*	• Not infiltrated<br>• <180°<br>• >180°<br><br>• Thrombosis (neoplastic / non-neoplastic)
	Splenic vein<br><br>*(<u>note</u>: vessels are considered to be not infiltrated when they are surrounded by a fat ring)*	• Not infiltrated<br>• <180°<br>• >180°<br><br>• Thrombosis (neoplastic / non-neoplastic)
Biliary ducts	Dilated	Yes/No; if yes:

		• diameter of left-sided ducts (mm) *[Numeric]*<br>• diameter of right-sided ducts (mm) *[Numeric]*<br>• diameter of common bile duct (mm) *[Numeric]*
Posterior lamina	Infiltration	• Yes<br>• No<br>• Uncertain (*[Free text]*)
Loco-regional diffusion	Stomach	Infiltration (yes/no)
	Spleen	Infiltration (yes/no)
	Duodenum	Infiltration (yes/no)
	Treitz	Infiltration (yes/no)
	Common bile duct	Infiltration (yes/no)
	Liver	Infiltration (yes/no)
	Other	*[Free text]*
Notes	*[Free text]*	
Peritoneal effusion	Peripancreatic effusion	Yes/No
	Supramesocolic effusion	Yes/No

	Pelvic effusion	Yes/No
	Supra- and submesocolic effusion	Yes/No
Peritoneal carcinomatosis	• No<br>• Yes (description *[Free text]*)	
Omentum	Infiltration	• Yes<br>• No<br>• Uncertain (*[Free text]*)

LOCO-REGIONAL LYMPHOADENOP-ATHIES	Yes/No; if yes:<br>• Peripancreatic<br>• Celiac<br>• Para-aortic<br>*(note: lymph nodes with long axis >5mm should be reported)*
DISTANT METASTASES	Yes/No
Liver	Yes/No; if yes:<br>Number of detectable lesions *[Numeric]*<br>For each target lesion (up to 2):<br>• site *[Liver segment]*

	• maximum diameter on axial images (mm) *[Numeric]*<br>• diameter perpendicular to maximum diameter (mm) *[Numeric]*<br>• structure *[Free text]*
Lung	Yes/No; if yes:<br>Number of detectable lesions *[Numeric]*<br>For each target lesion (up to 2):<br>• site *[Lung lobe, Lung segment]*<br>• maximum diameter on axial images (mm) *[Numeric]*<br>• diameter perpendicular to maximum diameter (mm) *[Numeric]*<br>Lymphangitic carcinomatosis (yes/no; if yes, site *[Free text]*)
Non-regional lymph nodes	Yes/No; if yes:<br>• site *[Free text]*<br>• maximum diameter on axial images (mm) *[Numeric]*<br>• diameter perpendicular to maximum diameter (mm) *[Numeric]*
Other organs (incl. bone)	• yes (site and type *[Free text]*)<br>• no

Acute pancreatitis	• No<br>• Yes, ongoing (report findings *[Free text]*)<br>• Yes, sequelae (pseudocysts *[Yes/no]*)
Pulmonary embolism	• No<br>• Yes (site and extent *[Free text]*)

INCIDENTAL FINDINGS	
Brain	*[Free text]*
Neck	*[Free text]*
Chest	*[Free text]*
Abdomen	*[Free text]*

CONCLUSIONS AND RECOMMENDATIONS	*[Free text]*

IMAGES

FIELD	DETAIL	ADMITTED VALUES

Key images	Key images	*[Images]*
